# Strategy of Surgical Management of Peripheral Neuropathy Form of Diabetic Foot Syndrome in Ghana

**DOI:** 10.1155/2014/185023

**Published:** 2014-07-24

**Authors:** W. M. Rdeini, P. Agbenorku, V. A. Mitish

**Affiliations:** ^1^Seventh Day Adventist Hospital, Kumasi, Ghana; ^2^Kwame Nkrumah University of Science & Technology, Kumasi, Ghana; ^3^Russian Peoples' Friendship University of Russia, Moscow, Russia

## Abstract

*Introduction.* Foot disorders such as ulceration, infection, and gangrene which are often due to diabetes mellitus are some major causes of morbidity and high amputation. * Aim.* This study aims to use a group of methods for the management of diabetic foot ulcers (DFU) in order to salvage the lower limb so as to reduce the rate of high amputations of the lower extremity. * Materials and Methods.* A group of different advanced methods for the management of DFU such as sharp debridement of ulcers, application of vacuum therapy, and other forms of reconstructive plastic surgical procedures were used. Data collection was done at 3 different hospitals where the treatments were given. * Results.* Fifty-four patients with type 2 diabetes mellitus were enrolled in the current study: females *n* = 37 (68.51%) and males *n* = 17 (31.49%) with different stages of PEDIS classification. They underwent different methods of surgical management: debridement, vacuum therapy (some constructed from locally used materials), and skin grafting giving good and fast results. Only 4 had below knee amputations. * Conclusion.* Using advanced surgical wound management including reconstructive plastic surgical procedures, it was possible to reduce the rate of high amputations of the lower limb.

## 1. Introduction

Diabetes mellitus is a condition characterized by a high blood glucose level occurring from inability of the pancreas to produce enough insulin or cells stop responding to the insulin that is produced [1]. It is a chronic disease that causes serious health complications including renal (kidney) failure, heart disease, stroke, and blindness. Symptoms include frequent urination, lethargy, excessive thirst, and hunger. There are several types of DM but under this study, the patients were under the type 2 DM. Type 2 DM occurs most often in people who are overweight and who do not exercise. The consequences of uncontrolled and untreated type 2 DM, however, are just as serious as those for Type 1 [1]. People with diabetes are usually older at presentation, usually above 30 years of age [2] and they may present with acute or chronic complications. The risk of a diabetic patient developing a foot ulcer may be as high as 25% [3] and can lead to considerable morbidity, amputation, and mortality [4]. Common etiologies are neuropathy, trauma, deformity, high plantar pressures, and peripheral arterial disease [5].

### 1.1. Epidemiology

For the past years globally, there has been an increase growth of the number of patients suffering from diabetes mellitus (DM) of which Ghana is no exception. From the data of the International Diabetic Federation in 2012, Ghana registered 354.02 per 1000 persons of 20–79 years amounting to about 3.16% of the population [6], but persons suffering from DM and not diagnosed range from ages 20 to 79 years which are 292.42 per 1000 persons, which form 2.61% population [6], out of which 12–15% of the patients have diabetic foot syndrome with ulcer defects [7, 8]. That figure is expected to double over the next decade. Over time many diabetic patients will develop the chronic complications such as retinopathy, nephropathy, peripheral neuropathy, and atherosclerotic vascular disease. Loss of a leg as a consequence of peripheral neuropathy or ischemia is from a patient's perspective one of the most feared of these complications is [9].

Diabetes is the most common medical condition leading to lower limb amputation and 85% of amputations are preceded by foot ulcers that fail to heal [3]. The main risks from diabetes are peripheral ischemia and neuropathy (both sensory and motor).

### 1.2. Aetiology of Diabetic Foot Problems

The main underlying risk factors for foot ulcers in diabetic patients are peripheral neuropathy and ischemia. For the purpose and scope of this research the emphasis would be highlighted on the neuropathy.

#### 1.2.1. Neuropathy

Prevalence of distal lower limb neuropathy affecting both type 1 and type 2 DM patients ranging from 30% to 50% has been reported in epidemiologic studies, a reason why more than 60% of diabetic patient foot ulcers are primarily due to underlying neuropathy [5, 10, 11]. The distal neuropathy of diabetes affects all components of the nervous system: sensory, motor, and autonomic, each of which contributes to foot ulcer development. Loss of nerve function correlates with chronic hyperglycaemia, as reflected in the mean level of glycosylated haemoglobin over time [7]. Ischemia of the endoneurial microvascular circulation induced by metabolic abnormalities from hyperglycaemia is believed to be the underlying mechanism for nerve deterioration [8].

#### 1.2.2. Motor Nerve Involvement

Imbalance of the long flexor and extensor tendons is caused by loss of neural supply to the intrinsic muscles of the foot. The classic high-arched foot and claw-toe deformity usually seen in about 50% of diabetics is induced by contraction of the more powerful flexors of the lower limb [12]. Hyperextension of the toes with resultant overriding of the metatarsal-phalangeal joints forces the metatarsal heads downward, thereby increasing their prominence further displacing the metatarsal fat pads distally, reducing the natural cushioning of the metatarsal heads. These mechanical changes increase plantar pressures inducing callus formation and underlying skin breakdown. Foot becomes wider and thicker; hence, patient's shoes no longer fit; this is caused by broadening of the foot from loss of the intrinsic muscles in combination with disruption of the normal bony relationships [13].

#### 1.2.3. Autonomic Neuropathy

Autonomic dysfunction of the foot from diabetic neuropathy results in loss of sweat and oil gland function. Anhidrosis leads to dry, fissured skin susceptible to bacterial invasion. Furthermore, loss of peripheral sympathetic vascular tone in the lower limb increases distal arterial flow and pressure, which, by damaging the capillary basement membrane, might contribute to peripheral oedema [6, 13]. Oedema increases the risk of foot ulceration by adding another element of minor trauma caused by wearing shoes that fit even more poorly as the oedema increases.

#### 1.2.4. Sensory Neuropathy

The concurrent loss of protective sensation in the foot is the cause of extensive motor and autonomic neural abnormalities [14]. Normally, if the foot developed a fissure or blister, or if bony structures change, patients would feel the discomfort and take appropriate corrective measures. Unfortunately, with onset of the peripheral neuropathy of diabetes, this protective response diminishes and can eventually disappear with progressive reduction in nerve function. This sequence of events allows patients to walk with apparent comfort on ever-deepening ulcers. The lack of pain lulls patients, and often physicians, into a false sense of security, a misguided “but it does not hurt; therefore it cannot be a serious problem” mentality [14].

### 1.3. Management

This involves testing ankle reflexes and vibration threshold, determining the degree of protective sensation, evaluating circulation, X-ray examinations, and bone scans for osteomyelitis [15]. Management includes good blood sugar control, avoiding pressure or trauma with good shoes or a special total contact cast, treating infection, improving circulation, and using topical therapy. Management of patients with ulcer defects in diabetic foot syndrome has been a serious problem in Ghana [6]. Different methods had been used including conventional methods. The late seeking for medical attention is as a result of early use of herbal preparations due to poverty and lack of education on the disease [16]. Sometimes bad judgment from the medical officer and the patients has also contributed to that effect. This nonsystemic management has led to high amputation of the lower limb for many of patients. For the past few years a lot of advanced methods were developed in the treatment of diabetic wounds [17–20]. Starting with the control of hyperglycemia, aggressive wound debridement, using vacuum therapy, using Versa jet system, and auto-skin grafting have contributed immensely to increase the effectiveness of management of diabetic wounds thereby avoiding the gross complications such as amputation in most cases. Some of the effective methods are expensive and not easily available for patients in Ghana including the wound vacuum system. In Ghana, a vacuum system locally manufactured which provides similar negative pressure wound therapy which is affordable has been developed [21].

### 1.4. Grading of DFU and Their Management

PEDIS is a method of classification of lesions in patients with diabetic foot syndrome. PEDIS stands for perfusion, extent (size), depth, infection, and sensation [22]. This is the classification used in this study.

## 2. Patients and Methods

### 2.1. Study Setting

The* Komfo Anokye Teaching Hospital (KATH)* located in Kumasi, the second largest in the country, is a tertiary health facility and a referral center serving persons in the Northern, Upper East and West, Brong Ahafo and Ashanti Regions. Currently the hospital has 1000 bed capacity. The hospital attends to about 679,050 annually consisting of in and out patients with a bed capacity of 1,000. Being a tertiary health facility, it is affiliated to the School of Medical Sciences (SMS) of Kwame Nkrumah University of Science and Technology (KNUST) [23].

*The Seventh Day Adventist (SDA) Hospital* is a district mission facility located in Kwadaso, a suburb of Kumasi. It was established in 1990 and has 84 patients' bed capacity [24].

*Effiduase District Hospital* is a district health facility in the Sekyere East District of the Ashanti Region; it was established in the 1950s to provide medical services to surrounding communities [25].

### 2.2. Data Collection

Data was collected based on patients' medical records at the three hospitals and the mode of treatment given to them by physicians and medical assistants. The work was based on the result of examinations and treatment of 54 patients with peripheral neuropathic form of diabetic foot syndrome (DFS) for the period January 1, 2011 till December 31, 2013.

### 2.3. Data Analysis

Data input was by MS excel and explained using qualitative analysis.

### 2.4. Ethical Clearance

Ethical clearance for this study was obtained from the KNUST School of Medical Sciences/KATH Committee on Human Research, Publication and Ethics, Kumasi.

### 2.5. Inclusion Criteria

Only patients who were diagnosed with type 2 diabetes were enrolled in the study.

### 2.6. Exclusion Criteria

Patients who had other types of diabetes mellitus were excluded from the study.

## 3. Results

### 3.1. Basic Data

In this series there were 54 DM patients: female *n* = 37 (68.5%), male *n* = 17 (31.5%); ages ranged 21–96 years; mean age = 54.9 years. The duration of DM from the day of diagnosis varied from few days to 26 years and the mean duration was 7.4 years. They were affected mostly on their feet and toes and other parts including the planter, dorsum, metatarsals, distal phalanges, and other parts below and above the ankle.

### 3.2. Categories of the DFU Patients and Their Management

#### 3.2.1. PEDIS 1

This involves wound without inflammation or purulence. Patients are usually treated with topical antibiotics. There were no patients within this group during the study period.

#### 3.2.2. PEDIS 2

**Figure 1:**
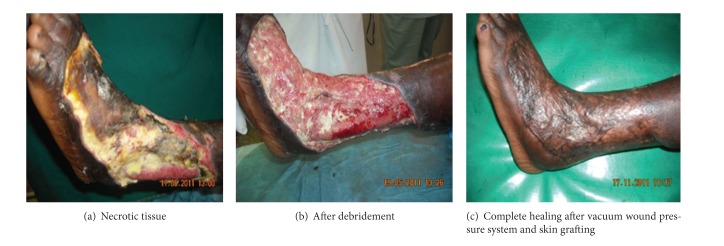
Example of PEDIS 2 DFU patients in the series.

There is purulence/erythema, pain, tenderness, and warmth/induration. There is cellulitis less than 2 cm around the ulcer and infection is limited to skin or subcutaneous tissue and usually not limb-threatening. This stage of diabetic ulcer included necrotizing inflammation, unhealed ulcers after amputation, gangrene, deep multiple chronic ulcers, leg cellulitis, wide deep necrotic ulcers, and septic wounds. Almost every patient had a multiple of these ulcerations. They were made up of 16 patients comprising 4 males and 12 females; their ages ranged 21–72 years living with their ulcers for at least 1 year and at most 15 years with a mean duration of 6.5 years. The ulcers were on their left or right foot, toes, and lateral and dorsal parts of their lower limbs. The ulcers were treated with debridement and excision of necrotic tissues and hyperkeratosis, wide excisions, and amputations of digits. All the patients were treated with povidone iodine daily dressings in combination with vaseline gauze and acetic acid dressings. Others also had daily foot baths. The wounds healed completely with 6 patients still under treatment (Table 1, Figure 1).

#### 3.2.3. PEDIS 3

**Figure 2:**
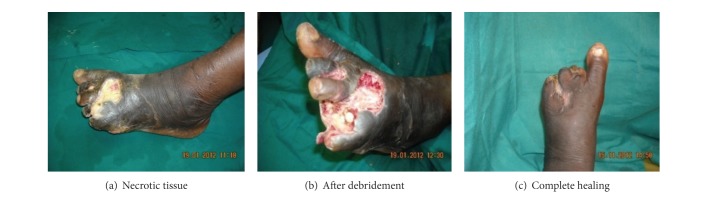
Example of PEDIS 3 DFU patients in the series.

Infection, cellulitis greater than 2 cm, streaking, deep tissue abscess, gangrene (may be life-threatening in some), muscle, tendon, joint, and bone may be involved. Eleven (11) patients had this stage of ulcer comprising of 3 males and 8 females. They were characterized by gangrene, necrotic ulcers, cellulitis, and osteomyelitis. They were treated with debridement, amputation of some parts of the foot, sequestrectomy, and dearticulation. Vacuum therapy and skin grafting were also done. Ulcers were managed with povidone iodine and normal saline daily dressings and metronidazole. One ulcer healed with contracture; four patients were still under treatment and the remaining 6 patients had their ulcers healing completely. The duration of patients' diagnosis of DM ranged from 10 to 26 years with a mean duration of 14.5 years; their ages ranged from 31 to 96 years. There was amputation involving the toes and metatarsal bones (Table 2, Figure 2).

#### 3.2.4. PEDIS 4

**Figure 3:**
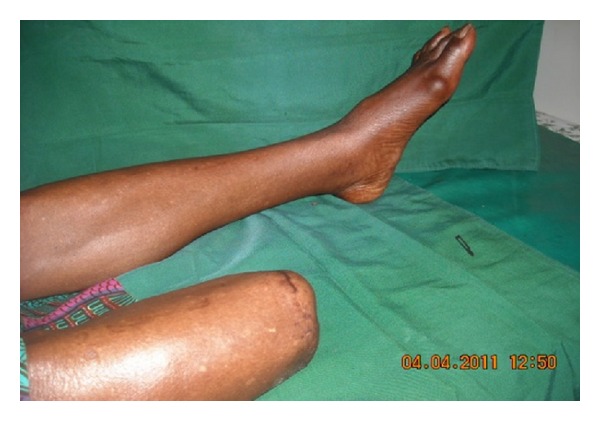
Example of PEDIS 4 DFU patients in the series: below knee amputation.

Infection with systemic toxicity, chills, fever, tachycardia, vomiting, acidosis, hypotension, hyperglycemia, and confusion are usually life-threatening as infection is severe. This grade of DM ulcer was characterized with DFS gangrene, necrosis, and cellulitis and it affected the foot above the ankle and the toes; there were 3 males and 2 females; their ages ranged 52–86 years. The duration of their DM ranged from 5 to 10 years with a mean duration of 4 years. Five patients were in this group and were treated by below knee amputations involving the tibia and fibula. Their ulcers were managed by daily dressing with povidone iodine and had no skin grafting. Four out of the 5 patients were discharged home with their amputated stumps well healed; one with ulcerated foot refused amputation and died a few days on admission (Table 3, Figure 3).

## 4. Discussion

The primary goal in the treatment of diabetic foot ulcers is to obtain wound closure. Presence of infection, severity, and vascularity determine the management of the foot ulcer [26, 27]. The first and most important procedure of ulcer therapy is debridement of all necrotic, callus and fibrous tissue [28–32] and this was applied to all degree of ulcers presented at the three hospitals. Unhealthy tissues of patients' ulcers were surgically debrided back to bleeding tissue to allow full visualization of the extent of the ulcer and detect underlying abscesses or sinuses. There was also excision of necrotic tissues and hyperkeratosis. It helped to reduce the rate of infection and provided an ideal healing environment by converting chronic wounds into acute; it also helped to reduce chronic inflammatory by-products as demonstrated by other authors in their studies [33–35].

Some researchers have reported toxification to healing of wounds by topical antiseptics such as povidone iodine [27, 29]; however in the current study povidone iodine was useful in wound dressing and facilitated healthy growth of ulcers in all patients.

Surgical drainage, deep debridement, or local partial foot amputations as done in some of the patients in the series are necessary adjuncts to antibiotic therapy of infections that are deep or limb-threatening; this was likewise proposed in some earlier studies by various authors [26, 36]. Hospitalization and surgical drainage becomes necessary when there is deep infection with abscess, cellulitis, gangrene, or osteomyelitis; foot infection and amputation as the major cause of hospitalization in diabetics have also been reported [37]. Also, in final healing of the foot ulcer especially in areas subject to exceedingly high plantar or shoe pressures, foot-sparing reconstructive procedures might be necessary [26, 38]. Antibiotics were subsequently tailored according to the clinical response of the patient and culture and sensitivity testing results. As in our current study, some other researches indicated that osteomyelitis is frequently present in patients with moderate to severe infections and requires aggressive bony incision of infected bone and joints followed by some weeks of culture-directed antibiotic therapy [38–41]. People with type 2 DM can control their condition with diet and oral medications. Insulin injections are sometimes necessary if treatment with diet and oral medication is not working [1]. Body exercise is also helpful [42].

Quality of life in patients with diabetic foot ulcers is low as the condition is related to comorbidity and in some cases fatalities [43, 44]. However, with the current study only one death was recorded as a result of patient's refusal to undergo surgery (amputation). Diabetic patients with foot ulcer compared to nondiabetics with foot ulcer have poor quality of life as well as survival rate [45]. However in the current study there was no such attempt to do any comparison with nondiabetic foot ulcers. Better management of patients could also be attained when there is proper grading of the patient, since it would help to determine the kind of treatment to be given out, since the likelihood of a patient to undergo surgery for amputation is highly dependent on the ulcer grade; thus, diabetic foot ulcers are complications of diabetes mellitus hence the earlier the detection implies avoidance of quick progression to the stage that may require amputation [46].

## 5. Conclusion

From the study, the use of advanced surgical wound management including reconstructive plastic surgical procedures made it possible to reduce the rate of amputations of the lower limb in patients. Early management of diabetic foot ulcers prevents complications which may require amputation of the affected person's limb as well as the quality of life.

## Figures and Tables

**Table 1:** PEDIS 2 DFU patients in the series.

Age (years)	Duration of DM (years)	Operation
21	4	Debridement, fixing of vacuum wound pressure system, and skin grafting
42	15	Debridement of necrotic tissues several times, excision of hyperkeratosis
63	4	Debridement
49	6	Wide excision of phlegmon from dorsal and planter aspects of the right foot and excision of necrotic tissues
62	5	Amputation of 1st toe of the right foot
64	12	Debridement, fixing of vacuum wound pressure system
52	5	Debridement
72	2	Debridement, fixing of vacuum wound pressure system
64	15	Debridement
72	10	Debridement, incision and drainage of abscess
42	0 (newly diagnosed)	Wound debridement
28	1	Debridement
36	8	Debridement
53	3	Debridement, fixing of vacuum wound pressure system
Unknown	8	Debridement

**Table 2:** PEDIS 3 DFU patients in the series.

Age (years)	Duration of DM (years)	Operation
52	26	Debridement, amputation of 2nd toe
40	12	Debridement, amputation of 1st toe of right foot; debridement repeated 3 times, and skin grafting
62	20	Debridement, amputation of phalanges 2nd and 4th toes of left foot
43	10	Debridement amputation of 3rd and 4th toes, incision of phlegmon of the right foot, and dearticulation of 2nd toe of the right foot
45	13	Debridement, incision of phlegmon
56	10	Debridement
31	12	Debridement, sequestrectomy
57	21	Debridement, amputation of left 3rd toe
96	11	Dearticulation
54	15	Debridement, digital dearticulation of 1st big toe of the right foot
56	10	Debridement

**Table 3:** PEDIS 4 DFU patients in the series.

Age (years)	Duration of DM (years)	Operation
75	0 (first diagnosed)	Debridement of dorsal and planter aspects of the right foot; patient refused amputation of necrotic toes; died a few days on admission
86	10	Below knee amputation
66	3	Below knee amputation
52	5	Below knee amputation
66	2	Below knee amputation
